# Hydro­nium 4-oxo-1,4-dihydro­pyridine-3-sulfonate dihydrate

**DOI:** 10.1107/S1600536809040641

**Published:** 2009-10-10

**Authors:** Zhi-Biao Zhu, Shan Gao, Seik Weng Ng

**Affiliations:** a College of Chemistry and Materials Science, Heilongjiang University, Harbin 150080, People’s Republic of China; b Department of Chemistry, University of Malaya, 50603 Kuala Lumpur, Malaysia

## Abstract

2-Hydroxy­pyridine when treated with concentrated sulfuric acid is sulfonated at the 3-position to yield the title hydrated salt, H_3_O^+^·C_5_H_4_NO_3_S^−^·2H_2_O. In the crystal structure, the cations, anions and uncoordinated water mol­ecules are linked by extensive O—H⋯O and N—H⋯O hydrogen bonds into a three-dimensional network. The crystal studied is a non-merohedral twin with a twin component of 36%.

## Related literature

For the treatment of non-merohedral twins, see: Spek (2003 ▶). For the cobalt salt of the anion, see: Zhu *et al.* (2007 ▶).
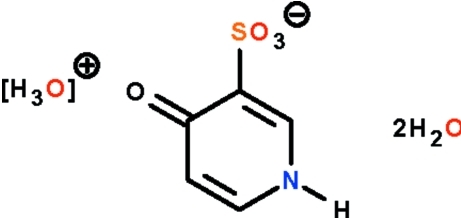


## Experimental

### 

#### Crystal data

H_3_O^+^·C_5_H_4_NO_4_S^−^·2H_2_O
*M* *_r_* = 229.21
Triclinic, 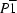

*a* = 6.6610 (8) Å
*b* = 7.4179 (10) Å
*c* = 10.0514 (11) Å
α = 90.922 (4)°
β = 94.543 (3)°
γ = 96.226 (4)°
*V* = 492.01 (10) Å^3^
*Z* = 2
Mo *K*α radiation
μ = 0.34 mm^−1^
*T* = 293 K
0.25 × 0.22 × 0.20 mm

#### Data collection

Rigaku R-AXIS RAPID IP diffractometer
Absorption correction: multi-scan (*ABSCOR*; Higashi, 1995 ▶) *T*_min_ = 0.856, *T*_max_ = 0.932
4711 measured reflections
2234 independent reflections
1719 reflections with *I* > 2σ(*I*)
*R*_int_ = 0.022

#### Refinement

*R*[*F* ^2^ > 2σ(*F* ^2^)] = 0.049
*wR*(*F* ^2^) = 0.170
*S* = 1.06
2234 reflections
152 parameters
13 restraints
H atoms treated by a mixture of independent and constrained refinement
Δρ_max_ = 0.65 e Å^−3^
Δρ_min_ = −0.59 e Å^−3^

### 

Data collection: *RAPID-AUTO* (Rigaku, 1998 ▶); cell refinement: *RAPID-AUTO*; data reduction: *CrystalClear* (Rigaku/MSC, 2002 ▶); program(s) used to solve structure: *SHELXS97* (Sheldrick, 2008 ▶); program(s) used to refine structure: *SHELXL97* (Sheldrick, 2008 ▶); molecular graphics: *X-SEED* (Barbour, 2001 ▶); software used to prepare material for publication: *publCIF* (Westrip, 2009 ▶).

## Supplementary Material

Crystal structure: contains datablocks global, I. DOI: 10.1107/S1600536809040641/xu2628sup1.cif

Structure factors: contains datablocks I. DOI: 10.1107/S1600536809040641/xu2628Isup2.hkl

Additional supplementary materials:  crystallographic information; 3D view; checkCIF report

## Figures and Tables

**Table 1:** Hydrogen-bond geometry (Å, °)

*D*—H⋯*A*	*D*—H	H⋯*A*	*D*⋯*A*	*D*—H⋯*A*
O1w—H11⋯O1	0.85 (1)	1.90 (1)	2.745 (4)	175 (5)
O1w—H12⋯O2^i^	0.85 (1)	2.50 (4)	2.983 (4)	116 (3)
O2w—H21⋯O1w	0.85 (1)	1.89 (1)	2.741 (4)	175 (6)
O2w—H22⋯O4^ii^	0.85 (1)	2.07 (2)	2.910 (4)	168 (6)
O3w—H31⋯O2^i^	0.86 (1)	2.12 (2)	2.951 (4)	165 (5)
O3w—H32⋯O2^ii^	0.85 (1)	2.07 (2)	2.898 (4)	162 (5)
O3w—H33⋯O3^iii^	0.85 (1)	2.18 (2)	2.936 (4)	147 (4)
N1—H1⋯O3^iv^	0.86 (1)	2.05 (2)	2.857 (4)	156 (5)

Symmetry codes: (i) 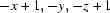; (ii) 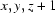; (iii) 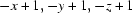; (iv) 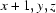.

**Fig. 1.**
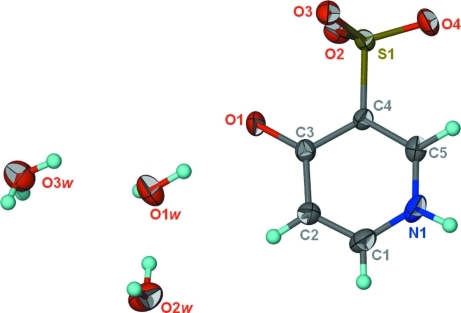
Thermal ellipsoid plot (Barbour, 2001) of [H3O][C5H4NO3S].2H2O at the 50% probability level; hydrogen atoms are drawn as spheres of arbitrary radius.

## supplementary crystallographic information

### Experimental

A 1:3 molar mixture of 4-hydroxypyridine and oleum (20%) was heated at 483 K for 3 h. Barium carbonate was added to the cool mixture until no more carbon dioxide evolved. Colorless crystals were isolated from the filtered solution after several days. CH&N elemental analysis. Calc. for C_5_H_10_NO_7_S: C 26.20, H 4.84, N 6.11%; found: C 26.36, H 4.48, N 6.11%.

### Refinement

Carbon-bound H-atoms were placed in calculated positions (C—H 0.93 to 0.97 Å) and were included in the refinement in the riding model approximation, with *U*(H) set to 1.2*U*(C). The water, oxonium and amino H-atoms were located in a difference Fourier map and refined with a distance restraint of O–H = N–H 0.85±0.01 Å. The temperature factors of some of the atoms could not be refined; as such, all temperature factors were tied.

The crystal is a non-merohedral twin as shown by *PLATON* (Spek, 2003).

### Crystal data

**Table:**

H_3_O^+^·C_5_H_4_NO_4_S^−^·2H_2_O	*Z* = 2
*M**_r_* = 229.21	*F*(000) = 240
Triclinic, *P*1	*D*_x_ = 1.547 Mg m^−^^3^
Hall symbol: -P 1	Mo *K*α radiation, λ = 0.71073 Å
*a* = 6.6610 (8) Å	Cell parameters from 3881 reflections
*b* = 7.4179 (10) Å	θ = 3.1–27.5°
*c* = 10.0514 (11) Å	µ = 0.34 mm^−^^1^
α = 90.922 (4)°	*T* = 293 K
β = 94.543 (3)°	Block, colorless
γ = 96.226 (4)°	0.25 × 0.22 × 0.20 mm
*V* = 492.01 (10) Å^3^	

### Data collection

**Table:**

Rigaku R-AXIS RAPID IP diffractometer	2234 independent reflections
Radiation source: fine-focus sealed tube	1719 reflections with *I* > 2σ(*I*)
graphite	*R*_int_ = 0.022
ω scans	θ_max_ = 27.5°, θ_min_ = 3.1°
Absorption correction: multi-scan (*ABSCOR*; Higashi, 1995)	*h* = −8→8
*T*_min_ = 0.856, *T*_max_ = 0.932	*k* = −9→9
4711 measured reflections	*l* = −13→13

### Refinement

**Table:**

Refinement on *F*^2^	Primary atom site location: structure-invariant direct methods
Least-squares matrix: full	Secondary atom site location: difference Fourier map
*R*[*F*^2^ > 2σ(*F*^2^)] = 0.049	Hydrogen site location: inferred from neighbouring sites
*wR*(*F*^2^) = 0.170	H atoms treated by a mixture of independent and constrained refinement
*S* = 1.06	*w* = 1/[σ^2^(*F*_o_^2^) + (0.0803*P*)^2^ + 0.6366*P*] where *P* = (*F*_o_^2^ + 2*F*_c_^2^)/3
2234 reflections	(Δ/σ)_max_ < 0.001
152 parameters	Δρ_max_ = 0.65 e Å^−^^3^
13 restraints	Δρ_min_ = −0.59 e Å^−^^3^

### Special details

**Table:**

Geometry. All e.s.d.'s (except the e.s.d. in the dihedral angle between two l.s. planes) are estimated using the full covariance matrix. The cell e.s.d.'s are taken into account individually in the estimation of e.s.d.'s in distances, angles and torsion angles; correlations between e.s.d.'s in cell parameters are only used when they are defined by crystal symmetry. An approximate (isotropic) treatment of cell e.s.d.'s is used for estimating e.s.d.'s involving l.s. planes.

### Fractional atomic coordinates and isotropic or equivalent isotropic displacement parameters (Å^2^)

**Table:**

	*x*	*y*	*z*	*U*_iso_*/*U*_eq_	
S1	0.68686 (13)	0.38798 (11)	0.16672 (8)	0.0280 (2)	
O1	0.6502 (4)	0.2383 (4)	0.4423 (2)	0.0363 (6)	
O2	0.5597 (4)	0.2165 (3)	0.1408 (2)	0.0365 (6)	
O3	0.5787 (4)	0.5226 (3)	0.2300 (2)	0.0350 (6)	
O4	0.7845 (4)	0.4562 (4)	0.0509 (2)	0.0402 (6)	
O1W	0.5750 (5)	0.0846 (4)	0.6825 (3)	0.0479 (7)	
H11	0.605 (9)	0.135 (5)	0.610 (3)	0.072*	
H12	0.523 (8)	−0.024 (3)	0.664 (4)	0.072*	
O2W	0.8700 (5)	0.1491 (5)	0.8890 (3)	0.0582 (8)	
H21	0.783 (7)	0.132 (7)	0.822 (4)	0.087*	
H22	0.855 (9)	0.249 (5)	0.928 (5)	0.087*	
O3W	0.2908 (5)	0.1387 (4)	0.9010 (3)	0.0540 (8)	
H31	0.310 (8)	0.028 (2)	0.887 (4)	0.081*	
H32	0.350 (7)	0.176 (5)	0.976 (2)	0.081*	
H33	0.338 (7)	0.204 (5)	0.839 (3)	0.081*	
N1	1.2314 (5)	0.3608 (5)	0.3510 (3)	0.0403 (7)	
H1	1.351 (3)	0.385 (7)	0.327 (5)	0.060*	
C1	1.1944 (6)	0.2958 (6)	0.4722 (4)	0.0405 (9)	
H1A	1.3022	0.2809	0.5342	0.049*	
C2	1.0013 (5)	0.2520 (5)	0.5047 (4)	0.0346 (8)	
H2	0.9795	0.2068	0.5887	0.042*	
C3	0.8321 (5)	0.2734 (4)	0.4136 (3)	0.0278 (6)	
C4	0.8828 (5)	0.3435 (4)	0.2858 (3)	0.0264 (7)	
C5	1.0802 (6)	0.3834 (5)	0.2586 (4)	0.0334 (7)	
H5	1.1100	0.4267	0.1752	0.040*	

### Atomic displacement parameters (Å^2^)

**Table:**

	*U*^11^	*U*^22^	*U*^33^	*U*^12^	*U*^13^	*U*^23^
S1	0.0305 (4)	0.0281 (4)	0.0249 (4)	0.0000 (3)	0.0025 (3)	0.0033 (3)
O1	0.0272 (12)	0.0504 (15)	0.0308 (12)	−0.0019 (11)	0.0066 (10)	0.0093 (11)
O2	0.0385 (13)	0.0313 (13)	0.0367 (13)	−0.0032 (11)	−0.0062 (11)	0.0012 (10)
O3	0.0367 (13)	0.0310 (12)	0.0389 (13)	0.0080 (11)	0.0072 (11)	0.0049 (10)
O4	0.0465 (16)	0.0461 (15)	0.0281 (12)	−0.0003 (13)	0.0082 (11)	0.0093 (11)
O1W	0.0621 (19)	0.0438 (16)	0.0346 (14)	−0.0083 (15)	0.0032 (14)	0.0057 (11)
O2W	0.0456 (17)	0.066 (2)	0.062 (2)	0.0103 (17)	−0.0055 (16)	−0.0101 (16)
O3W	0.063 (2)	0.0457 (17)	0.0525 (18)	0.0037 (15)	0.0025 (16)	0.0059 (13)
N1	0.0244 (14)	0.0515 (19)	0.0452 (18)	0.0016 (14)	0.0100 (14)	−0.0040 (14)
C1	0.0287 (18)	0.050 (2)	0.043 (2)	0.0081 (16)	−0.0009 (16)	−0.0059 (17)
C2	0.0309 (18)	0.043 (2)	0.0300 (16)	0.0057 (15)	0.0013 (14)	0.0019 (14)
C3	0.0269 (15)	0.0281 (15)	0.0274 (15)	−0.0018 (13)	0.0031 (13)	0.0003 (12)
C4	0.0233 (14)	0.0282 (16)	0.0275 (15)	0.0017 (12)	0.0030 (12)	−0.0005 (12)
C5	0.0325 (17)	0.0332 (17)	0.0347 (17)	0.0002 (15)	0.0087 (15)	−0.0017 (13)

### Geometric parameters (Å, °)

**Table:**

S1—O4	1.449 (2)	O3W—H33	0.854 (10)
S1—O2	1.457 (2)	N1—C5	1.341 (5)
S1—O3	1.459 (3)	N1—C1	1.348 (5)
S1—C4	1.762 (3)	N1—H1	0.855 (10)
O1—C3	1.268 (4)	C1—C2	1.358 (5)
O1W—H11	0.850 (10)	C1—H1A	0.9300
O1W—H12	0.854 (10)	C2—C3	1.419 (5)
O2W—H21	0.854 (10)	C2—H2	0.9300
O2W—H22	0.851 (10)	C3—C4	1.445 (4)
O3W—H31	0.855 (10)	C4—C5	1.367 (5)
O3W—H32	0.853 (10)	C5—H5	0.9300
			
O4—S1—O2	113.46 (15)	N1—C1—H1A	119.7
O4—S1—O3	112.83 (16)	C2—C1—H1A	119.7
O2—S1—O3	111.76 (16)	C1—C2—C3	121.7 (3)
O4—S1—C4	106.31 (16)	C1—C2—H2	119.2
O2—S1—C4	106.22 (15)	C3—C2—H2	119.2
O3—S1—C4	105.54 (15)	O1—C3—C2	123.1 (3)
H11—O1W—H12	108.9 (17)	O1—C3—C4	122.2 (3)
H21—O2W—H22	108.9 (17)	C2—C3—C4	114.7 (3)
H31—O3W—H32	109.7 (17)	C5—C4—C3	120.9 (3)
H31—O3W—H33	109.4 (17)	C5—C4—S1	119.7 (3)
H32—O3W—H33	109.9 (17)	C3—C4—S1	119.3 (2)
C5—N1—C1	121.5 (3)	N1—C5—C4	120.5 (3)
C5—N1—H1	116 (3)	N1—C5—H5	119.7
C1—N1—H1	122 (3)	C4—C5—H5	119.7
N1—C1—C2	120.7 (4)		
			
C5—N1—C1—C2	−0.4 (6)	O2—S1—C4—C5	124.1 (3)
N1—C1—C2—C3	−0.3 (6)	O3—S1—C4—C5	−117.1 (3)
C1—C2—C3—O1	−178.0 (4)	O4—S1—C4—C3	179.9 (3)
C1—C2—C3—C4	0.4 (5)	O2—S1—C4—C3	−58.9 (3)
O1—C3—C4—C5	178.7 (3)	O3—S1—C4—C3	59.8 (3)
C2—C3—C4—C5	0.3 (5)	C1—N1—C5—C4	1.0 (6)
O1—C3—C4—S1	1.8 (5)	C3—C4—C5—N1	−1.0 (5)
C2—C3—C4—S1	−176.6 (2)	S1—C4—C5—N1	175.9 (3)
O4—S1—C4—C5	2.9 (3)		

### Hydrogen-bond geometry (Å, °)

**Table:**

*D*—H···*A*	*D*—H	H···*A*	*D*···*A*	*D*—H···*A*
O1w—H11···O1	0.85 (1)	1.90 (1)	2.745 (4)	175 (5)
O1w—H12···O2^i^	0.85 (1)	2.50 (4)	2.983 (4)	116 (3)
O2w—H21···O1w	0.85 (1)	1.89 (1)	2.741 (4)	175 (6)
O2w—H22···O4^ii^	0.85 (1)	2.07 (2)	2.910 (4)	168 (6)
O3w—H31···O2^i^	0.86 (1)	2.12 (2)	2.951 (4)	165 (5)
O3w—H32···O2^ii^	0.85 (1)	2.07 (2)	2.898 (4)	162 (5)
O3w—H33···O3^iii^	0.85 (1)	2.18 (2)	2.936 (4)	147 (4)
N1—H1···O3^iv^	0.86 (1)	2.05 (2)	2.857 (4)	156 (5)

Symmetry codes: (i) −*x*+1, −*y*, −*z*+1; (ii) *x*, *y*, *z*+1; (iii) −*x*+1, −*y*+1, −*z*+1; (iv) *x*+1, *y*, *z*.
